# Simultaneous bilateral posterior ischemic optic neuropathy secondary to giant cell arteritis: a case presentation and review of the literature

**DOI:** 10.1186/s12886-018-0994-9

**Published:** 2018-12-12

**Authors:** Anas Mohammad Albarrak, Yousef Mohammad, Sajjad Hussain, Sufia Husain, Taim Muayqil

**Affiliations:** 1 Department of Internal Medicine, College of Medicine, Prince Sattam Bin Abdulaziz University, Alkharj, Saudi Arabia; 2 Department of Internal Medicine, Neurology division, King Khalid University Hospital, Riyadh, Saudi Arabia; 3 Department of Medical Imaging, King Saud University / King Khalid University Hospital, Riyadh, Saudi Arabia; 4 Department of Pathology and Laboratory Medicine, King Khalid University Hospital, College of Medicine, King Saud University, Riyadh, Saudi Arabia; 5 Neurology division, Department of Medicine, College of Medicine, King Saud University, Riyadh, Saudi Arabia

**Keywords:** Giant cell arteritis, Blindness, Headache, Posterior ischemic optic neuropathy, Neuro-ophthalmology

## Abstract

**Background:**

This report highlights a rare case of simultaneous bilateral blindness due to posterior ischemic optic neuropathy. Typically, ophthalmic involvement in giant cell arteritis is monocular or sequential ischemia of the anterior portion of the optic nerve, and less frequently simultaneous.

**Case presentation:**

An 80-year-old Saudi male came with a history of simultaneous bilateral vision loss 5 days prior to presentation. The exam showed dilated non-reactive pupils, no light perception in both eyes, and normal fundus exam. C-reactive protein and erythrocyte sedimentation rate levels were high Magnetic resonance imaging and magnetic resonance angiography of the brain showed a right posterior optic nerve lesion and absence of flow in both ophthalmic arteries respectively. A left temporal artery biopsy confirmed giant cell arteritis.

**Conclusion:**

The presentation of GCA can be atypical and patients may present with simultaneous blindness. Bilateral simultaneous PION does not exclusively occur in a post surgical setting, emphasizing the importance of decreasing the threshold of suspicion of similar cases to avoid further neurological complications.

## Background

Giant cell (temporal) arteritis (GCA) is a chronic vasculitis of large and medium-sized vessels, which can affect individuals over 50 years of age [1] and is the most common systemic vasculitis [2]. Typically, patients may present with a new headache, jaw claudication and/or monocular vision loss [3]. In comparison to anterior ischemic optic neuropathy (AION), posterior ischemic optic neuropathy (PION) is an uncommon form of optic nerve ischemia that can occur secondarily to GCA [4]. Here, we describe an elderly patient who presented with simultaneous bilateral loss of vision due to posterior ischemic optic neuropathies and biopsy proven GCA. This case outlines the importance of rapid recognition of GCA in the setting of an uncommon presentation as early management can prevent devastating and irreversible complications.

## Case report

We report a case of an 80-year old hypertensive and diabetic Saudi male referred to our center after developing sudden bilateral painless visual loss five days earlier. There was a history of a bilateral temporal headache that had started a year earlier. The headache was more prominent on the left side, mild to moderate in severity, and stabbing in nature. It used to occur on average once a week and would spontaneously resolve over several seconds. However, the frequency had increased in the months preceding visual loss, occurring almost daily. There was no report of any previous episodes of diplopia, transient visual loss, jaw claudication, myalgia, constitutional symptoms, motor or sensory symptoms.

**Fig. 1:**
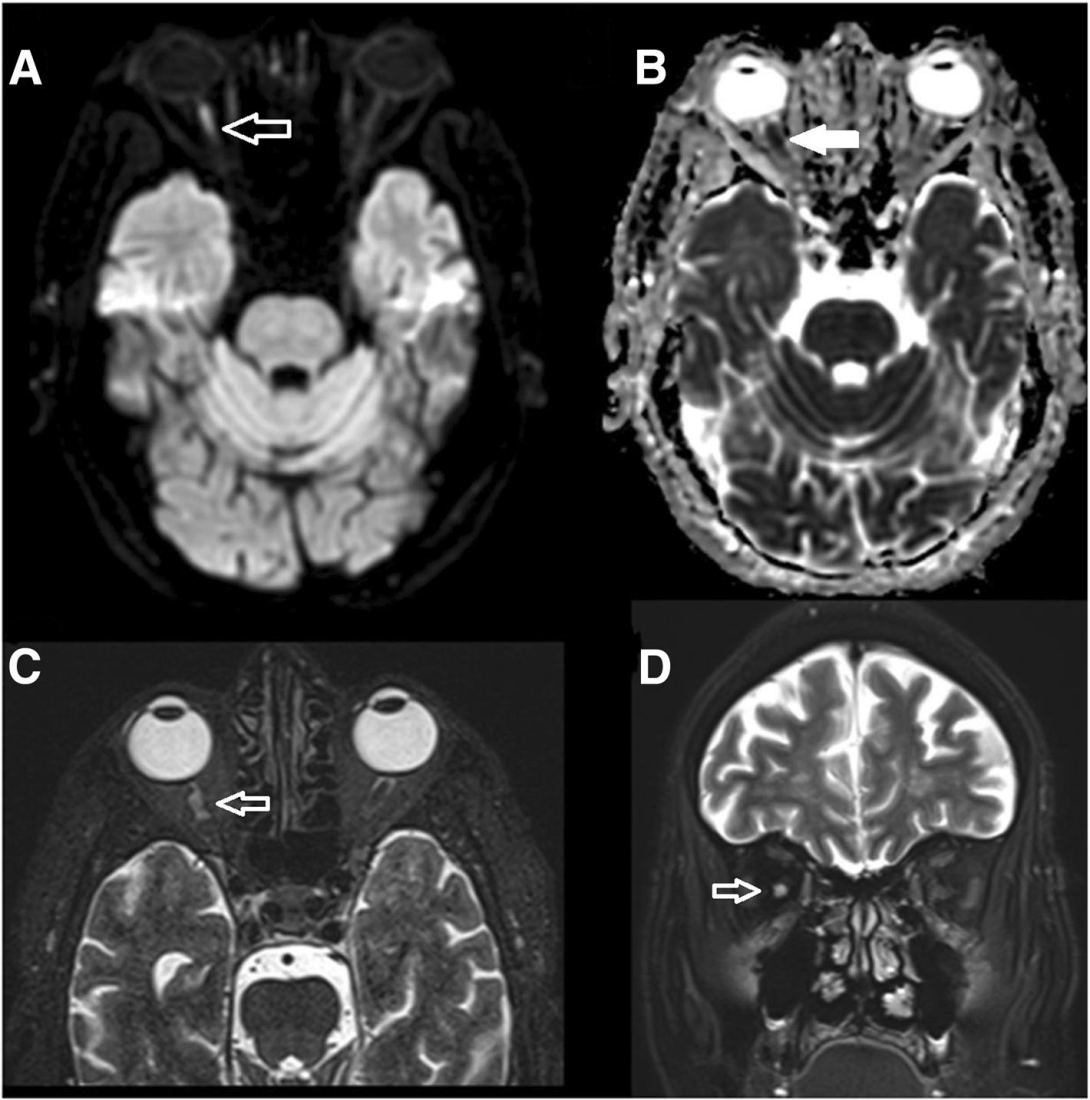
**a** Axial diffusion weighted images. **b** ADC maps, **c** high resolution T2 weighted images and **d** coronal T2 weighted images. Arrows showing true diffusion restriction in the right optic nerve with T2 signal hyperintensity

**Fig. 2:**
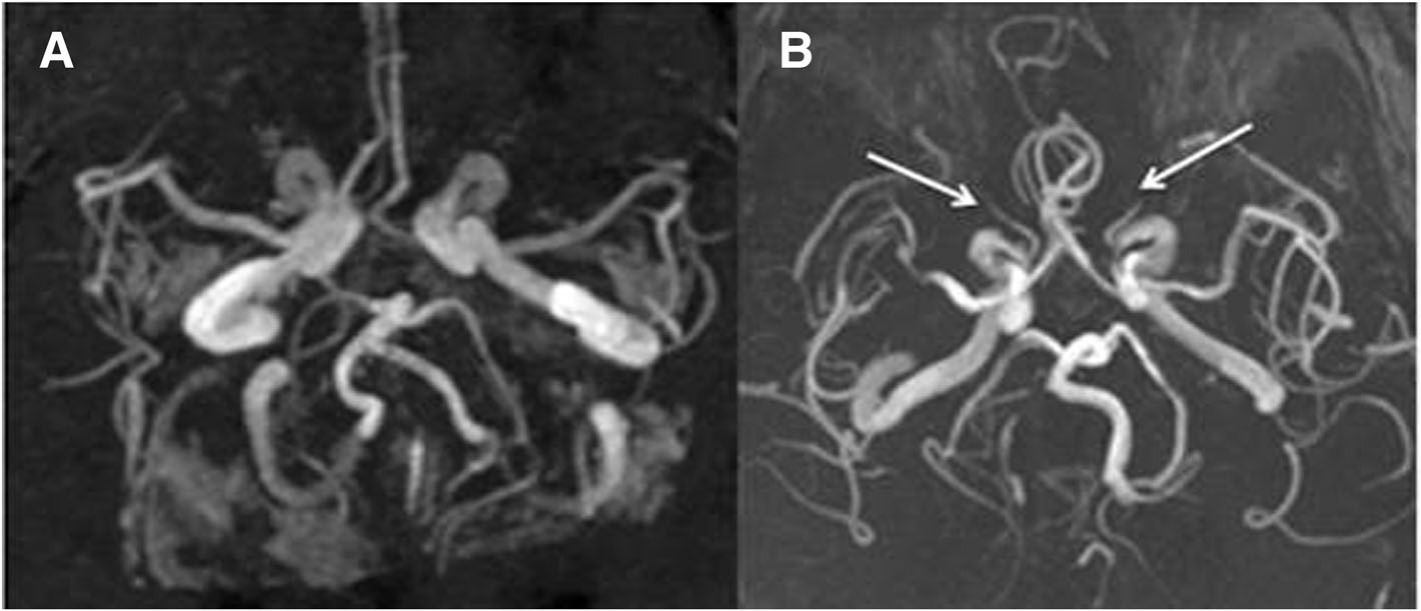
MRA of the patient (**a**) versus a normal individual (**b**) with arrows indicating severe narrowing in ophthalmic arteries in the patient bilaterally

Examination showed normal blood pressure, heart rate, and temperature. He was unable to perceive light in both eyes, and the pupils were bilaterally dilated, seven millimeters each, with no reaction to light. Fundoscopy showed normal appearing discs and retina. Ocular movements were full. The motor, sensory and coordination examination was normal. The C-reactive protein (CRP) upon admission was 132 mg/L and the erythrocyte sedimentation rate (ESR) was 40 mm/hr. A magnetic resonance imaging (MRI) of the brain was done (Fig. 1) and it showed a lesion in right optic nerve suggesting acute ischemia. The ophthalmic arteries were not visualized bilaterally by contrast magnetic resonance angiography (MRA) (Fig. 2). The clinical impression was of a bilateral Posterior Ischemic Optic Neuropathy (PION) due to giant cell arteritis (GCA).

**Fig. 3:**
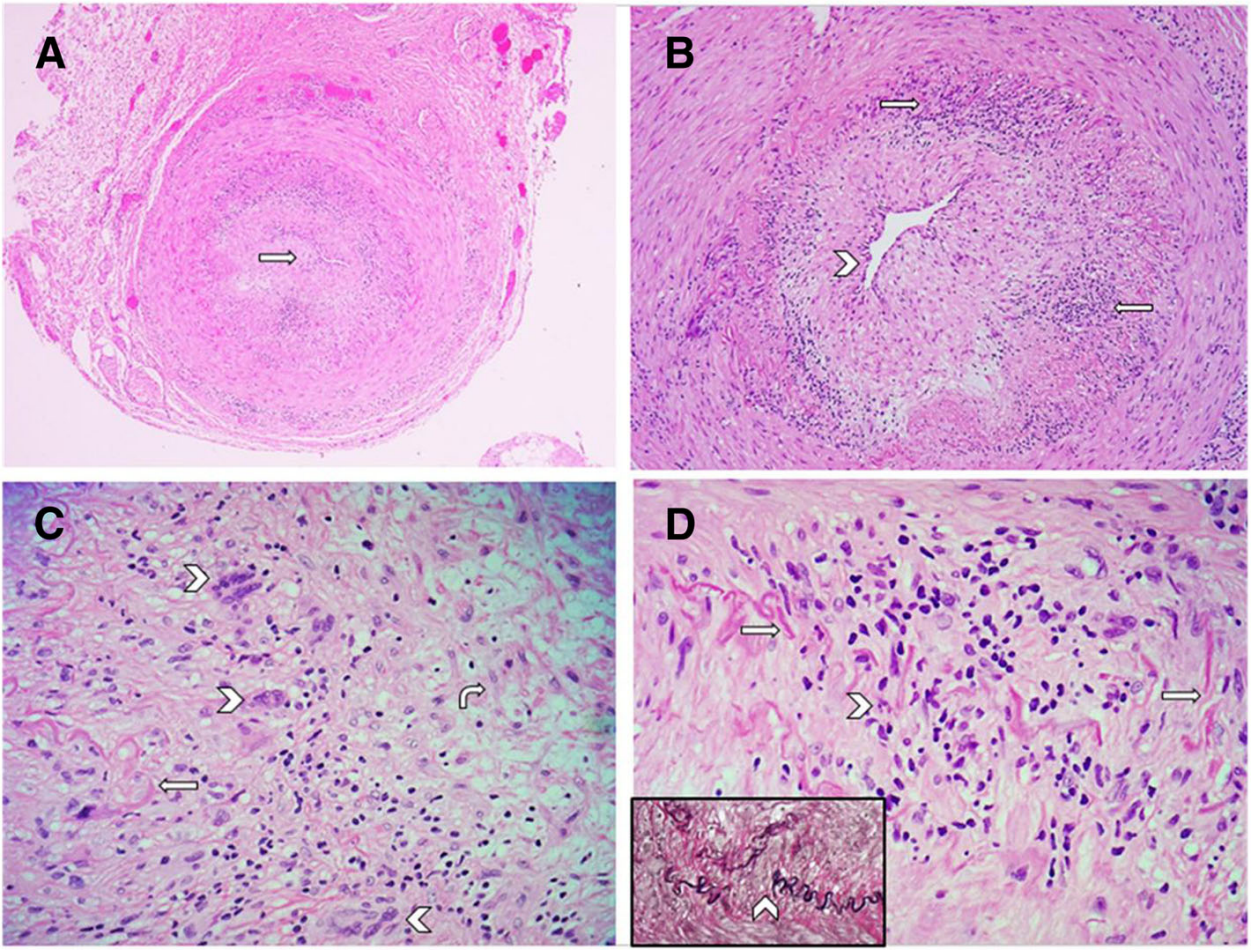
Photomicrograph of a cross section of temporal artery shows (**a**) almost total occlusion of the vascular lumen (arrow) due to fibrointimal proliferation and arteritis (Hematoxylin and eosin stain; original magnification × 100.). **b** Infiltration of the arterial intima and media by patchy moderately dense lymphohistiocytic inflammatory cells (arrows). The arterial lumen is reduced to a narrow slit-like channel (arrowhead)(Hematoxylin and eosin stain; original magnification × 200.) **c** High magnification shows mononuclear inflammatory cell infiltrate mixed with scattered multinucleated giant cells (arrowhead) in the vessel wall. Fragmentation of the internal elastic lamina (arrow) and fibrointimal proliferation and edema (curved arrow) of the arterial intima is also shown (Hematoxylin and eosin stain; original magnification × 400.) **d** High magnification shows a lymphohistiocytic inflammatory cell infiltrate (arrowhead) destroying and disrupting the internal elastic lamina (arrow). (Hematoxylin and eosin stain; original magnification × 400.) Inset: shows elastic tissue stain highlighting the fragmented internal elastic lamina (arrowhead). (Elastic Van Gieson stain; original magnification × 400

The patient was started on intravenous Methylprednisolone 1000 mg for five days then shifted to daily prednisolone 60 mg orally. The vision did not improve but the headache improved significantly after a few days. A 2 cm segment of the left temporal artery was biopsied and the pathological findings confirmed giant cell arteritis (Fig. 3).

## Discussion

Here we present a patient of Arab ethnicity who developed bilateral blindness after suffering from a simultaneous onset bilateral PION and a biopsy proven GCA. GCA is a granulomatous, medium to large vessel, inflammatory disease that can have devastating ischemic consequences to the eye [5, 6]. It is considered a relatively unusual occurrence in Arabs [7] and is more commonly encountered in Caucasian ethnicity [8]. Its incidence is most marked among white individuals of Scandinavian descent [9, 10], with one large Norwegian study finding an increasing incidence between 1972 and 1992 that subsequently plateaued. Women are more likely to be affected than men and the typical patient is in the 8th decade of life [5, 9]. The typical symptoms of GCA include headache, scalp tenderness, jaw claudication, and loss of vision [3]. Headache is a common symptom in GCA and occurs in more than two-thirds of patients [11]. Visual impairments of varying degrees occur in one or both eyes in about 25 to 50% of GCA patients [4, 12, 13]. PION from GCA is uncommon (Table 1), the visual loss is most frequently the result of anterior ischemic optic neuropathies (AION), followed by central or branch retinal arterial occlusions [4, 8, 14]. In a prospective series of 170 patients with GCA, AION occurred in 81.2% of ocular ischemic cases, while PION occurred unilaterally in 5.9% and bilaterally in one patient [4]. Approximately 10% of visual impairment was from CRAO [4]. PION results from ischemia involving the retrobulbar part of the optic nerve [4], and is classified by etiology as non-arteritic, arteritic or surgical (perioperative) [4]. Typically, PION presents with monocular visual loss when non-arteritic and sequential visual loss when arteritic. Simultaneous bilateral visual loss is usually encountered in the perioperative type of PION [15] where perfusion abnormalities to the eyes occur [16]. In contrast to AION, the optic disc examination is normal in the acute setting of PION, with the development of optic atrophy on follow up assessments appearing about 6 weeks after the event [17]. Findings on MRI are either normal or show hyper-intense signals on diffusion restricted images, while enhancement of the optic nerve with contrast can be seen in arteritic ischemic optic neuropathies [18].

**Table 1:** Reports of posterior ischemic optic neuropathy secondary to giant cell arteritis

	Number of patients	Number of cases	Clinical details
Liozon et al. 2001[12]	174 GCA 147 Biopsy proven GCA 48 Ophthalmic GCA	2 (PION)	Both described as bilateral and recovered
Hayreh et al. 1998 [4]	170 Biopsy proven GCA 85 Ophthalmic GCA	6 (PION)	One described as Bilateral
Garrity et al. 2017 [8]	32 Biopsy proven ophthalmic GCA	2 (PION)	African American cohort
Danesh-Meyer et al. 2005 [21]	34 Biopsy proven ophthalmic GCA	2 (PION)	One had PION on one side and AION on the other side that was described as bilateral at presentation
Liu et al. 1994 [22]	45 biopsy proven ophthalmic GCA	2 (PION)	One had PION on one side and AION on the other side that was simultaneous onset
Aiello et al. 1993 [23]	327 GCA 245 Ophthalmic diagnosis 204 biopsy proven	1 (PION)	
Chaudhry et al. 2007 [7]	102 Suspected ophthalmic GCA 7 biopsy proven	1 (PION)	
Sadda et al. 2001[15]	72 PION patients	6 (Arteritic)	None simultaneous due to arteritis
Hayreh 2004 [17]	43 PION patients	12 (Arteritic)	

The current patient presented with a history of headache for one year that culminated in bilateral visual loss from optic nerve ischemia. The differential diagnosis for a presentation of acute optic neuropathy with headache includes arteritic ischemic optic neuropathy, infections (cat-scratch, syphilis), inflammatory (para-infectious, multiple sclerosis, systemic autoimmune, paraneoplastic, and sarcoidosis). GCA in the current scenario presented fairly typically, readily distinguishing it from other conditions. The ESR and CRP were high which suggested an inflammatory process and abnormal signals were seen on the MRI suggesting infarction.

Simultaneous and sudden complete blindness of both eyes due to PION is an unusual presentation in GCA. Bilateral visual loss is usually due to the sequential onset of AION, CRAO, or PION in any combination [4, 15]. One cohort that looked at a small subgroup of simultaneous onset bilateral ischemic optic neuropathies found examination findings of different ages in each eye, raising the possibility that patients may not become aware of visual loss until it involves the other eye [4]. Thus, an important consideration in the current case is defining how simultaneous was the onset. Our patient witnessed the visual loss occur while he was awake, and was found to have symmetrical exam findings when assessed at the referring center on the day of onset, and again at our center 5 days later with no clear disc abnormalities. There was diffusion restriction involving the right optic nerve with absence of both ophthalmic arteries by MRA. It was not surprising to find asymmetrical findings on MRI as PION changes may not always seen on MRI [19], and our patient did not have imaging done until one week after the event reducing the possibility of witnessing acute changes. Asymmetry in the observed MRI signals of bilateral simultaneous onset post surgical PION have also been described [20].

## Conclusions

In this report we presented a patient who was clinically and histologically diagnosed as GCA. IV steroids were started upon his arrival five days from symptom onset, with dramatic improvement in headache, however, no improvement in vision occurred as is the case with optic nerve ischemia secondary to GCA [21]. Given the yearlong history of headache, this case emphasizes the importance of early recognition of headache disorders among elderly; the threshold of suspecting GCA in elderly patients should be very low. This case highlights a rarely identified ocular presentation of GCA, it is important to identify this early in order to start treatment to avoid devastating and potentially permanent ocular or systemic complication. When bilateral PION occurs simultaneously in a non-surgical setting in an older individual, then the possibility of GCA as an etiology should be considered high.

## Abbreviations

AION: Anterior ischemic optic neuropathy
CRP: C-reactive protein
ESR: Erythrocyte sedimentation rate
GCA: Giant cell arteritis
MRI: Magnetic resonance imaging
MRA: Magnetic resonance angiography
PION: Posterior ischemic optic neuropathy
